# Melanocortin-4 receptor and leptin as genes for the selection of superior Madrasin cattle

**DOI:** 10.14202/vetworld.2021.3224-3228

**Published:** 2021-12-31

**Authors:** Budi Utomo, Rimayanti Rimayanti, Indah Norma Triana, Amaq Fadholly

**Affiliations:** 1 Division of Veterinary Reproduction, Faculty of Veterinary Medicine, Universitas Airlangga, Surabaya, 60115, Indonesia

**Keywords:** leptin, Madrasin, melanocortin-4 receptor

## Abstract

**Background and Aim:**

The genetic improvement of cattle through livestock section is based on quantitative, qualitative, and molecular characteristics. This study examined polymorphisms of the melanocortin-4 receptor (MC4R) and leptin genes as a reference for the selection of superior breeds in Madrasin cattle.

**Materials and Methods:**

The leptin and MC4R genes of Madrasin cattle were amplified using polymerase chain reaction (PCR); then, restriction fragment length polymorphism of the leptin gene was performed using the restriction enzyme BsaA1, at site 2793 with ACGT point position.

**Results:**

The leptin gene was divided into three bands, namely, AA with one fragment (522 bp), CG with two fragments (441 bp and 81 bp), and AG with three fragments (522 bp, 441 bp, and 81 bp). The MCR-4 gene was divided into three bands, namely, 493 bp, 318 bp, and 175 bp.

**Conclusion:**

The MC4R and leptin genes can act as molecular markers for growth traits in Madrasin cattle and can be used to genetically optimize and improve growth. The GG allele of the MC4R gene and the AA allele of the leptin gene can be used in Madrasin cattle.

## Introduction

Because of its great variety of plant and animal species, including cattle genetic resources, Indonesia has very high biodiversity (mega biodiversity). Several breeds of cattle, both local and exotic, are well distributed within the country. The Madura cattle, a cross between the wild Bos and *Bos javanicus* breeds, are popular because of their uniform shape. The deoxyribonuclease acid (DNA) of the Madura breed is a mixture of the Zebu cow and bull. Meanwhile, in several areas on Madura Island, artificial insemination is being used aggressively to cross local and Limousine cattle to produce offspring known as Limura; this community is known for crossing local and exotic breeds in hopes of producing highly productive calves [1,2].

Melanocortin-4 receptor (MC4R) is the primary gene responsible for regulating food intake and energy balance. MC4R is a pair of G protein receptors expressed in the hypothalamic nucleus, as it also plays an important role in the regulation of homeostasis. The protein regulates behavior related to food intake and energy expenditure; it is responsible for the obesity response to leptin in vertebrates, including humans [3]. The gene also produces synthesized leptin from adipose tissue. This hormone plays an important role in controlling body weight, feed intake, and energy balance. Furthermore, polymorphisms in the MC4R and leptin genes affect body weight and intramuscular fat deposition in supporting efforts, which can increase the productivity of Limura cattle [4]. However, this type of study is lacking in Madrasin cattle.

This study examined polymorphisms of MC4R and leptin genes as a reference for the selection of superior breeds in Madrasin cattle.

## Materials and Methods

### Ethical approval

The study was approved by Animal Care and Use Committee of Veterinary of Medicine Faculty, Universitas Airlangga (reference number: 1.KE.200.03.2021).

### Study period and location

This study was conducted from March 2021 to September 2021 at Center of Veterinary, Bali, Indonesia.

### Sample collection

Semen samples were obtained from Madrasin bulls, ranging in age from 2-4 years. Before semen collection, the preputial hairs were clipped, and the orifice was washed with clean water and then dried with clean paper towel to minimize contamination. DNA samples were obtained from the semen.

### DNA extraction

A total of 10 million sperm cells/μL were extracted through the spin column method, using theQiAamp DNA Mini Kit (Qiagen, Canada), according to the manufacturer’s instructions. The total DNA was then stored in a freezer at −20°C until further analysis.

### PCR amplification

Using the duplex PCR method, the total extracted sperm DNA was further amplified at a volume of 25 μL, which contained 12.5 μL GoTaq Green Master Mix (Promega, USA), 1 μL primer F (10 pmol), 1 μL primer R (10 pmol), 8.5 μL nuclease-free water (Promega), and 2 μL DNA samples. The two pairs of primers used were forward 5’- GTCGGGCGTCTTGTTCATC-3’ and reverse 5’-GCTTGTGTTTAGCATCGCGT-3’ [5]. Moreover, amplification was conducted using a PCR (Applied Biosystem, USA) machine, which consisted of pre-denaturation, denaturation, annealing, and extension at 94°C, 94°C, 58°C, and 72°C for 5 min, and 30, 30, and 30 s, with 45 repeated cycles, respectively. The cycle ended with a final extension of 72°C for 10 min, followed by a hold at 4°C

Amplification of the exon 3 region of the leptin gene used forward L1 (5’-GTCTGGAGGCAAAGGGCAGAGT-3’). Pre-denaturation, denaturation, annealing, and extension at 94, 94, 64, and 72°C were conducted for 5 min, and 30, 30, and 30 s, with 45 repeated cycles, respectively. The cycle ended with a final extension of 72°C for 10 min, accompanied by a hold at 4°C.

### PCR-restriction fragment length polymorphism (PCR-RFLP)

PCR products (amplified specific MC4R DNA fragments [493 bp] and leptin [580 bp] genes) were digested with HpyCH4IV (restriction enzyme) and single-nucleotide polymorphisms (SNPs) g.1133C > G. The RFLP process was carried out at a total volume of 20.3 μL, consisting of 2.8 μL DDW, 2 μL 10× buffer, 0.5 μL HpyCH4IV, and 15 μL PCR product, which were further incubated at 37°C for 5 h through a multi-heater. The digestion results were electrophoresed on 4% agarose gel, and staining was conducted by adding 1 μL ethidium bromide to 50 μl of 1× Tris/Borate/EDTA solution. The final result was observed visually using an ultraviolet transilluminator.

### Electrophoresis

The PCR products were analyzed through electrophoresis on 1.5% agarose gel with SYBR dye (Invitrogen S7563, USA). Molecular markers (Invitrogen) measuring 100 bp were also added to determine the size of the PCR products. Furthermore, the results were observed using a gel documentation system (Bio-Rad, USA).

### Statistical analysis

The allele and genotype frequencies of each sample were calculated following the measurement of unidirectional pattern variance.

## Results

### Amplification of the leptin and MC4R genes

**Figure-1:**
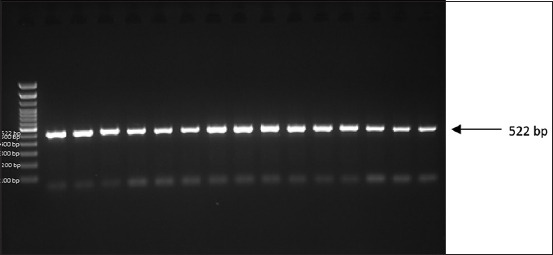
Leptin gene amplification on Madrasin cattle.

**Figure-2:**
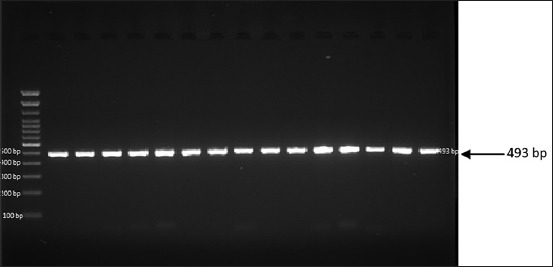
Melanocortin-4 receptor gene amplification of Madrasin cattle.

To amplify the leptin and MC4R genes, DNA material was isolated from 15 samples of Madrasin cattle blood. The electrophoretic results showed that the genes amplified to 522 (leptin) and 493 (MC4R) bp, as shown in Figures-1 and 2, respectively. These results indicated that the leptin and MC4R genes can be amplified through PCR.

### PCR-RFLP of the leptin and MC4R genes

**Figure-3:**
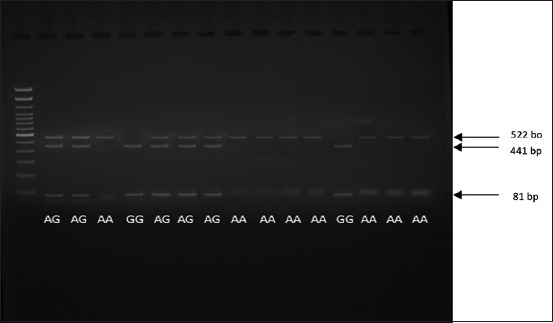
Leptin gene polymerase chain reaction-restriction fragment length polymorphism with restriction enzyme BsaA1.

RFLP or DNA cutting of the leptin gene was conducted at site 2793, through the BsaA1 (restriction enzyme) and AC|GT cut point position. We obtained 15 genetic samples of the hormone, divided into three bands of 522, 441, and 81 bp. Based on Figure-3, the leptin gene had three genotypes, namely, AA, GG, and AG, with one, two, or three fragments (corresponding to 522 bp; 441 and 81 bp; 522, 441, and 81 bp). Furthermore, the restriction enzymes recognized the leptin gene at the cutting site because the DNA sequence did not undergo any mutations. The results of the PCR-RFLP for the leptin gene are shown in Figure-3.

**Figure-4:**
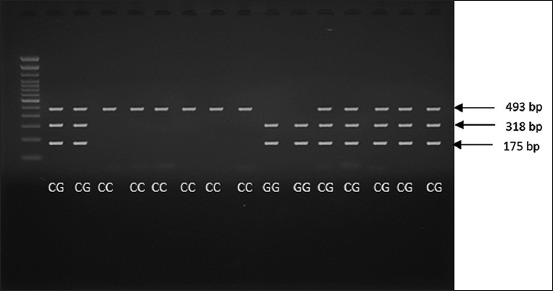
Melanocortin-4 receptor gene polymerase chain reaction-restriction fragment length polymorphism with HpyCH4IV restriction enzyme.

Fifteen samples of the MC4R gene were obtained, divided into three bands of 493, 318, and 175 bp. Moreover, the GG, CG, and CC genotypes originated from the cattle whose blood was analyzed using the PCR-RFLP method. In addition, the GG and CG genotypes had two and three DNA fragments at 175 and 318 bp and at 175, 318, and 493 bp, respectively, whereas the CC genotype had one large fragment at 493 bp (Figure-4).

## Discussion

Genotyping is the process of identifying genotypes in individuals through DNA markers at a specific locus within the genome [6]. Genotyping results are often used to describe the distribution of alleles within a population based on observations of genetic diversity. Genotyping is also used to determine the population balance, according to the Hardy–Weinberg equilibrium. Based on the DNA markers of a target gene, results can be used to describe the genotype of each animal under investigation [7]. Genotypic determinations are often conducted using PCR-RFLP.

Polymorphisms in several portions of the leptin gene have the potential to be used for molecular selection; these polymorphisms are reportedly associated with body and carcass weights, milk protein content, backfat thickness, and rib-eye areas [8-10]. Polymorphism in intron 2 is also associated with milk production [11,12], body and carcass backfat thickness, and empty period [13-15]. Meanwhile, polymorphisms in intron 2 do not correlate with adult weight in Iraqi local and Fries Holstein (exotic) cattle. The leptin gene polymorphism in exon 2 is further associated with carcass weight, chest circumference, thick back fat, milk production, and feed intake [16-18]. In exon 3, polymorphisms are correlated with body and carcass weights, marbling score, and non-return rate [19]. Furthermore, mutations in the exon portion of the gene cause changes in certain amino acids, affecting livestock productivity. For example, a mutation in the g.1233C > T position within the Lep/BamHI gene causes arginine to be exchanged with cysteine. It is associated with chest circumference in Anatolian Black cattle. A mutation in the g.1863C > T position in the Lep/HphI gene (exon 2) also causes alanine to be changed to valine but is not associated with productivity; this lack of effect on productivity is due to the two amino acids being located in the b helix of leptin. Although alanine and valine are similar types of protein (non-polar aliphatic R group), they do not induce changes in the receptor structure [20]. Mutations at positions g.820C > T and g.2270A > G (Lep/Sau3AI) in Sistani (*Bos indicus*) cattle are associated with weaning weight. However, they should be further examined in *B. indicus* breeds in Indonesia (e.g., the Ongole, Aceh, and Madura species). Moreover, mutations at g.252T > A, g.1127A > T, g.1233C > T, and g.14911 (Lep/ClaI, Lep/BspDI, Lep/BamHI, and Lep/MspI), as well as their effect on the productivity of *B. indicus* and *B. javanicus* cattle, also require further examination [21,22].

Leptin gene utilization was carried out by identifying the genotype in a livestock population, since selected cattle should meet the standards of good farming practices, for the expression of their genetic potential. Our results showed that the leptin gene had three genotypes, namely, AA, GG, and AG with one, two, or three fragments. The genotypic data were associated with livestock productivity (assuming a large number of cows were evaluated). In beef cattle, when the heterozygous genotype (AB) leads to the best average performance, that animal should be used for the feedlot. The high performance of this genotypic attribute is caused by the effect of heterosis (hybrid vigor), which indicates that the capabilities of offspring are higher than those of their parents [23]. Therefore, stocking homozygous cattle (AA and BB) are necessary to have sources for the production of heterozygous (AB) breeds. When two similar average (AA and AB) and one low (BB) performance genotypes are obtained, the AB and BB individuals should be culled; cattle with AB genotypes are still excluded and not used as breed sources, due to having poor performance B allele traits. However, AA individuals are utilized to produce feeders.

MC4R gene plays an important role in regulating food intake and body weight in native Korean cattle (brown, striped, and black) [24]. Due to mutations, the gene encoding this receptor contributes to obesity. The MC4R gene acts as an adrenocorticotropic hormone, as well as an alpha-beta- and gamma-melanocyte-stimulating hormone (MSH), which plays an important role in energy homeostasis and somatic growth, or as a receptor on the heptapeptide nucleus. Somatic growth has an important role in cattle development and is controlled by a complex system. The genes carrying out somatic growth are responsible for cattle development after birth. The MC4R gene acts on energy homeostasis mediated by alpha-MSH [25]. Our data showed that GG and CG genotypes had two and three DNA fragments, whereas the CC genotype had one large fragment. MC4R-C1069G is an SNP in various cattle breeds due to being associated with fat thickness, as well live and carcass weights [26]. Furthermore, cattle with the GG genotype have higher economic characteristics and better growth than GC and CC genotypes. In Korean cattle, the GG genotype of the Luxi breed also has better economic features than those with the CC genotype. The live and carcass weights, as well as the backfat thickness of cattle with the GG genotype, were higher than those of the CG and CC genotypes [26]. Similar results have also been noted in adult Hanwoo cattle, with higher live and body weight values attributed to the GG genotype. In addition, the GG genotype of the PO Kebumen breed has a higher birth length (66.34±6.73 cm) than the CC (65.43±7.21 cm) and GC (59.47±5.77 cm) genotypes [27,28].

## Conclusion

The use of the MC4R and leptin genes as molecular markers was applied to optimize and improve the genetic quality of Madrasin cattle growth. Moreover, the GG (MC4R) and AA (leptin) alleles of both genes were found to be good sources for breeding. The MC4R and leptin genes can act as molecular markers for growth traits in Madrasin cattle and can be used to optimize genetically and improve growth. The GG allele of the MC4R gene and the AA allele of the leptin gene were found in Madrasin cattle.

## Authors’ Contributions

BU: Designed and performed the study. BU and RR: Provided materials and critical reviews. BU, RR, INT, and AF: Literature search and manuscript preparation. All authors read and approved the final version of the manuscript.
